# Caffeine exacerbates seizure-induced death via postictal hypoxia

**DOI:** 10.1038/s41598-023-41409-6

**Published:** 2023-08-29

**Authors:** Antis G. George, Alyssa Federico, Renaud C. Gom, Sydney A. Harris, G. Campbell Teskey

**Affiliations:** 1 Cumming School of Medicine, Hotchkiss Brain Institute, University of Calgary, Calgary, AB T2N 4N1 Canada; 2 Department of Cell Biology and Anatomy, University of Calgary, Calgary, AB Canada; 3 Department of Clinical Neurosciences, University of Calgary, Calgary, AB Canada; 4 Department of Clinical Neurosciences, Cumming School of Medicine, University of Calgary, 3330 Hospital Drive N.W. Calgary, Alberta, T2N 4N Canada; 5 Alberta Children’s Hospital Research Institute, Cumming School of Medicine, University of Calgary, Calgary, AB Canada

**Keywords:** Diseases of the nervous system, Neuroscience, Physiology

## Abstract

Sudden unexpected death in epilepsy (SUDEP) is the leading epilepsy-related cause of premature mortality in people with intractable epilepsy, who are 27 times more likely to die than the general population. Impairment of the central control of breathing following a seizure has been identified as a putative cause of death, but the mechanisms underlying this seizure-induced breathing failure are largely unknown. Our laboratory has advanced a vascular theory of postictal behavioural dysfunction, including SUDEP. We have recently reported that seizure-induced death occurs after seizures invade brainstem breathing centres which then leads to local hypoxia causing breathing failure and death. Here we investigated the effects of caffeine and two adenosine receptors in two models of seizure-induced death. We recorded local oxygen levels in brainstem breathing centres as well as time to cessation of breathing and cardiac activity relative to seizure activity. The administration of the non-selective A_1_/A_2A_ antagonist caffeine or the selective A_1_ agonist N6-cyclopentyladenosine reveals a detrimental effect on postictal hypoxia, providing support for caffeine modulating cerebral vasculature leading to brainstem hypoxia and cessation of breathing. Conversely, A_2A_ activation with CGS-21680 was found to increase the lifespan of mice in both our models of seizure-induced death.

## Introduction

Sudden unexpected death in epilepsy (SUDEP) is one of the most common causes of death associated with epilepsy. SUDEP accounts for 17% of all epilepsy-related deaths and nearly 50% of deaths as a direct result of epilepsy in individuals with chronic refractory epilepsy^1–3^. The standardized mortality rate in young adults (20–40 years) makes SUDEP the second leading neurological cause of death in terms of years of potential life lost, behind stroke^4–7^. Several mechanisms have been proposed for SUDEP including cardiac, arousal and neurotransmitter dysfunction and obstructive apneas^8–10^. Although the underlying mechanism remains elusive, data from epilepsy monitoring units have refined our understanding of SUDEP and provide evidence that SUDEP is ultimately caused by a failure to breathe soon after a seizure ends^11^.

While the pathophysiological underpinnings of postictal respiratory failure remains elusive, emerging data highlight a candidate cause^12–17^. In 2016, Farrell and colleagues demonstrated that seizures in the forebrain induce postictal vasoconstriction leading to hypoperfusion and brain tissue hypoxia in both rodents and humans and is severe enough to cause behavioural dysfunction^12,16^. George and colleagues (2023) recently tested the neurovascular theory of SUDEP and determined that localized brainstem postictal hypoxia was a key driver in seizure-induced mortality in both acute and chronic mouse models, thus providing the mechanistic antecedent to the collapse of cardiorespiratory function which characterizes SUDEP^17^.

Further probing the neurovascular consequence of seizures, Phillips, and colleagues (2019) determined that acute caffeine and its metabolites reduced baseline hippocampal oxygen levels and worsened seizure-induced hypoxia^18^. Caffeine is the most widely consumed psychoactive drug in the world with an average intake around 300 mg/day^19,20^. Currently, limited caffeine consumption has been advised for people with epilepsy by some authors^21–23^. However, patient education on caffeine avoidance is not standard which may impart harm on those with epilepsy and at greatest risk for SUDEP^20^.

The present study tested the neurovascular theory of SUDEP and utilized pharmacological approaches to examine the effects of caffeine and A_1_ and A_2A_ receptors on two mouse models of seizure-induced death while simultaneously recording brain tissue oxygen levels in the hippocampus and brainstem breathing centers. Acutely, using intrahippocampal kainic acid and chronically using *Kcna1*^−*/*−^ mice, we found that caffeine significantly accelerated time to death in both models, indicating that caffeine consumption could be a modifiable risk factor in SUDEP pathophysiology.

## Results

### Acute caffeine, theophylline and N6 accelerate time to death whereas CGS-21680 delays it

**Figure 1:**
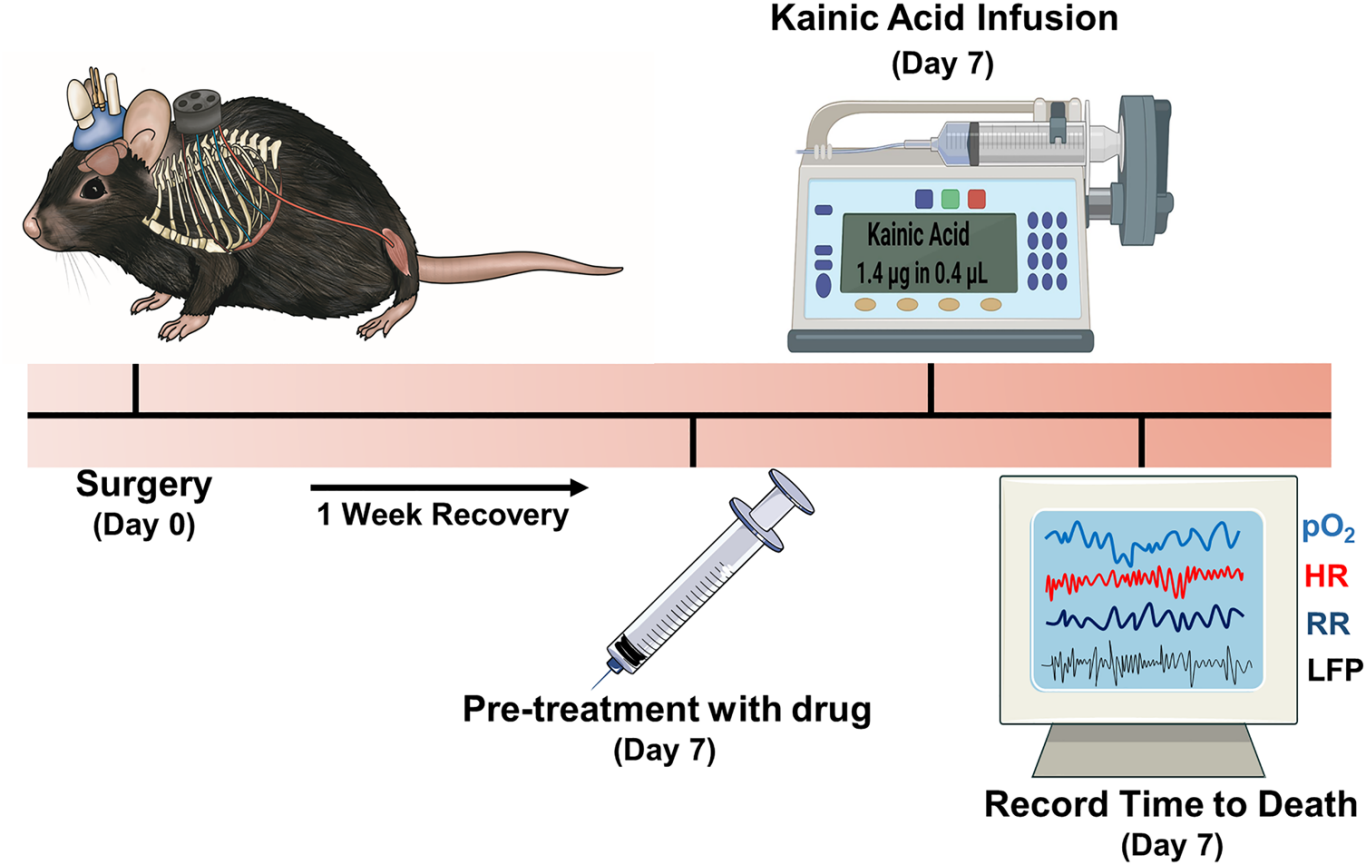
Acute experimental timeline. Male C57BL/6J mice on P50 underwent brain implantation surgery and diaphragmatic surgery (Day 0). Mice recovered for one week before beginning terminal experiment (Day 7). Mice were injected with either caffeine, theophylline, N6-Cyclopentyladenosine (N6) or CGS- 21,680, 30 min prior to infusion of kainic acid. Physiological parameters including heart rate, respiratory rate, brain partial pressure of oxygen and local field potential were recorded until death.

**Figure 2:**
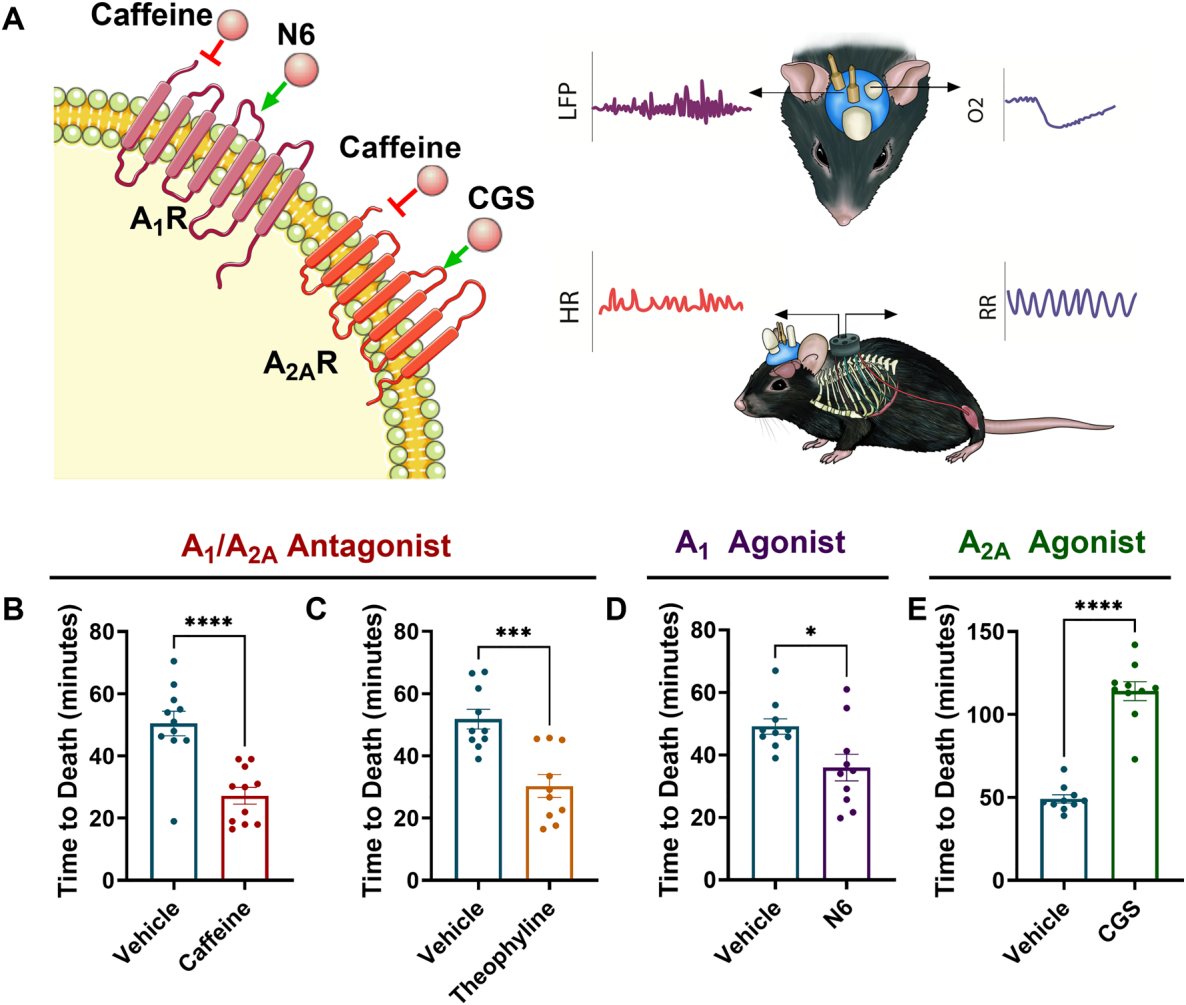
Drug targets of adenosine A_1_ and A_2A_ receptor subtype. (**A**) Model schematic: following the injection of vehicle or drug, cardiorespiratory (HR: heart rate/ RR respiratory rate), oxygen and LFP signals were measured. electromyograph (EMG) electrode wires record respiratory activity directly from diaphragm and electrodes record cardiac activity. A bipolar electrode in the brainstem records local field potential (LFP) in the pre-Bötzinger complex (PBC) and an oxygen sensing optode in the contralateral PBC records absolute oxygen. Mean time to death in male C57BL6/J mice treated with drugs targeting adenosine receptor subtypes. (**B**) Vehicle mice (n = 11) mean time to death was 50.44 ± 3.95, Caffeine mice (n = 11) mean time to death was 27.21 ± 2.65 (t_20_ = 4.87, p =  < 0.0001****). (**C**) Vehicle mice (n = 10) mean time to death was 51.80 ± 3.17, Theophylline (n = 10) mean time to death was 30.32 ± 3.67 (t_18_ = 4.42, p = 0.0003***). (**D**) Vehicle mice (n = 10) mean time to death was 49.12 ± 2.44, N6 (n = 10) mean time to death was 35.95 ± 4.27 (t_18_ = 2.67, p = 0.0154*). (**E**) Vehicle mice (n = 10) mean time to death was 19.12 ± 2.44, CGS-21680 (n = 10) 114.0 ± 5.72 (t_18_ = 10.42, p =  < 0.0001****). Histobars represent mean ± SEM.

Having previously established an acute model of seizure-induced death in mice^14^, we sought to screen caffeine and other drugs of interest to determine their relationship to SUDEP (Fig. 1). We first sought to determine if adenosine receptor modulation had an effect on time to seizure-induced death. Here we tested agonists and antagonists on the two primary adenosine receptors expressed in the brain: A_1_ and A_2A_. After intrahippocampal administration of the chemoconvulsant kainic acid in C57BL/6J mice, all mice were monitored using cardiorespiratory physiology and brain tissue oxygen to establish time to death defined as terminal apnea detected by diaphragmatic EMG^17^. All mice displayed several bouts of bilateral tonic–clonic seizures with intervening behavioral recovery. On average, mice that received vehicle died of seizure-induced terminal apnea 49.78 ± 1.30 min after intrahippocampal administration of kainic acid (Fig. 2). Mice that were treated with caffeine a non-selective A_1_/A_2A_ antagonist died sooner than vehicle mice with a mean time to death of 27.21 ± 2.65 min. We then examined theophylline, a metabolite of caffeine and a non-selective A_1_/A_2A_ antagonist. Theophylline displayed a similar temporal profile to caffeine, having their time to death accelerated with a mean time to death of 30.32 ± 3.67 min. To determine if caffeine was interacting with the A_1_ receptor to influence time to death, we used the selective A_1_ agonist N6-cyclopentyladenosine (N6). Mice that were treated with N6 had their time to death accelerated with a mean time to death of 35.95 ± 4.27 min. We next determined if caffeine’s influence on time to death was mediated by the A_2A_ receptor. To this end we used CGS-21680, a selective A_2A_ agonist. Mice that received CGS-21680 displayed increased survival compared to vehicle, caffeine and N6 treated mice with a mean time to death of 114.0 ± 5.72 min. Our data provide evidence that caffeine accelerated time to death likely independent of the A_1_ receptor as the use of the A_1_ agonist N6 mirrored the effects of caffeine, a non-selective antagonist. Using CGS-21680 as a probative tool, caffeine is likely interacting at the A_2A_ receptor to accelerate time to death from hypoxia.

### Acute caffeine reduces baseline brainstem oxygen

**Figure 3:**
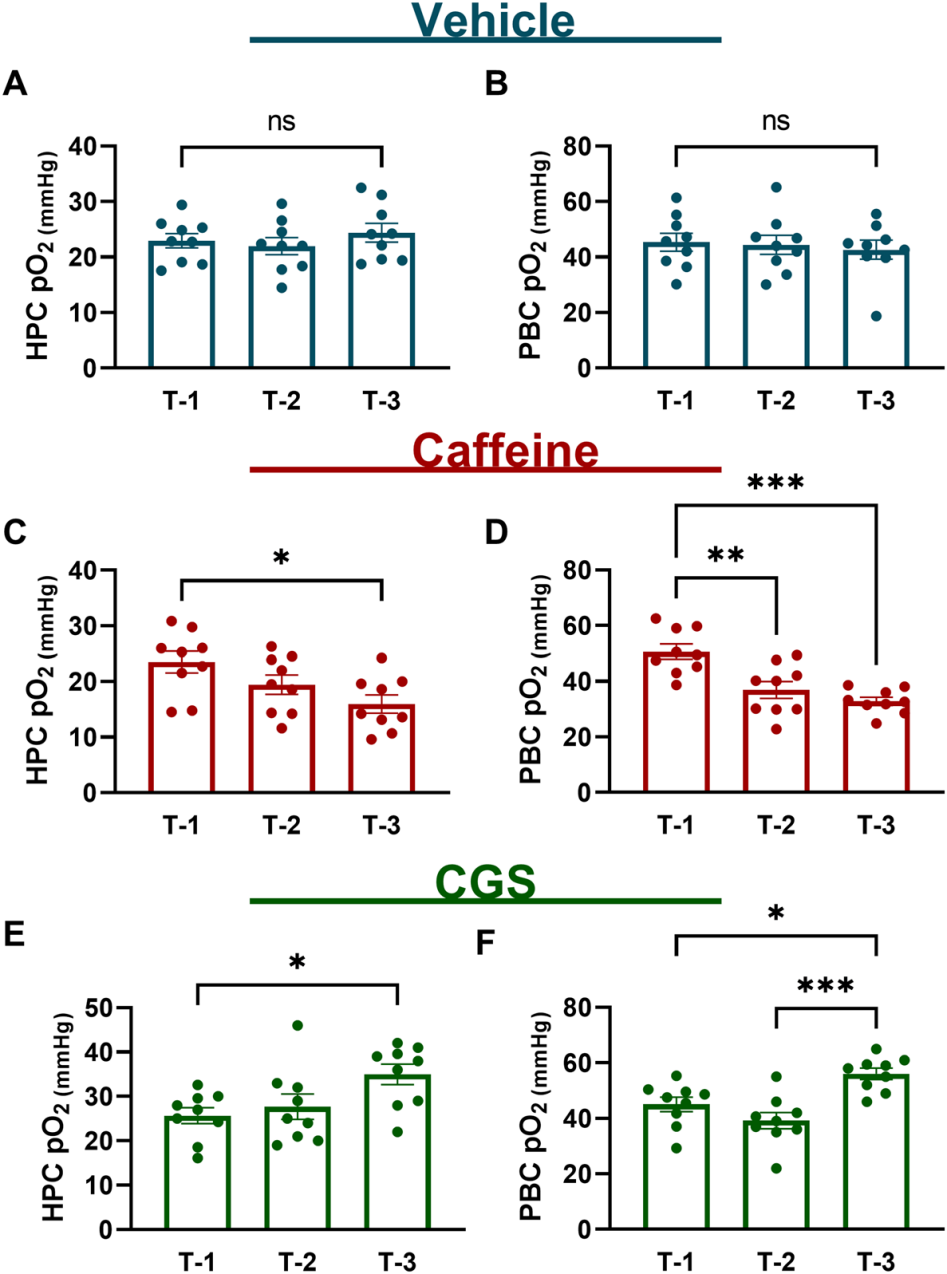
Caffeine reduced baseline brainstem oxygen. Mean partial pressure of oxygen (mmHg) pre-injection of drug or vehicle baseline (T-1); 10-min post-injection of drug or vehicle baseline (T-2); Mean 30-min post-injection of drug or vehicle baseline (T-3). (**A**,**B**) Hippocampus (HPC) and pre-Bötzinger complex (PBC) oxygen differences were not statistically significant in vehicle mice (n = 9). (**C**) HPC caffeine: (n = 9), T-1 = 23.51 ± 1.93, T-2 = 19.46 ± 1.72, T-3 = 15.95 ± 1.62, (ANOVA F (2, 24) = 4.567, p = 0.0209). (**D**) PBC caffeine: T-1 = 50.64 ± 2.72, T-2 = 36.93 ± 3.02, T-3 = 32.85 ± 1.43, (ANOVA F (2, 24) = 13.90, p =  < 0.0001***). (**E**) HPC CGS-21680: (n = 9), T-1 = 25.68 ± 1.80, T-2 = 27.67 ± 2.84, T-3 = 34.96 ± 2.31, (ANOVA F (2, 24) = 4.281, p = 0.026). PBS CGS-21680: T-1 = 45.03 ± 2.62, T-2 = 39.18 ± 2.98, T-3 = 56.04 ± 2.02, (ANOVA F (2,24) = 11.09, p = 0.0004***). Histobars represent mean ± SEM.

A previous study examining rat hippocampal oxygen levels demonstrated that caffeine significantly reduces baseline oxygen and exacerbated seizure induced hypoxia^18^. Here we monitored brain tissue oxygenation in the hippocampus (HPC) and the pre-Bötzinger complex (PBC), a respiratory nucleus located in the ventral medulla responsible for the generation of rhythmic inspiratory breathing movements in C57BL/6J mice^24^. We measured oxygen levels at three time points prior to intrahippocampal kainic acid infusion to examine the influence of adenosinergic agonists and antagonists on physiological oxygen values in the HPC and PBC of the brainstem (Fig. 3). We examined baseline oxygen levels at pre-injection of drug or vehicle (T-1), 10-min post-injection of drug or vehicle (T-2) and 30-min post-injection of drug or vehicle (T-3). Vehicle treated mice (n = 11) did not have significantly different oxygen values in the hippocampus or brainstem at any time point. Mice that were treated with caffeine (n = 11) displayed significant reductions between T-1 baseline and T-3 in the hippocampus showing a 38.15% difference in partial pressure of oxygen. Caffeine also significantly lowered oxygen values in the brainstem. There was a 31.33% difference between T-1 and T-2 and a 42.64% difference in oxygen between T-1 and T-3 baseline. Mice treated with the A_2A_ agonist CGS-21680 (n = 11) had significant increase in HPC oxygen between T-1 and T-3 with a difference of 30.61%. In the PBC, there was a 21.80 difference between T-1 and T-3 and a 24.34% difference between T-2 and T-3. Our data indicate that caffeine, an A_2A_ antagonist, decreases baseline oxygen values in the forebrain and brainstem. In opposition, CGS-21680, an agonist at the A_2A_ receptor, raises baseline oxygen in both brain regions.

### Acute caffeine administration accelerates time to terminal hypoxia, apnea and death

**Figure 4:**
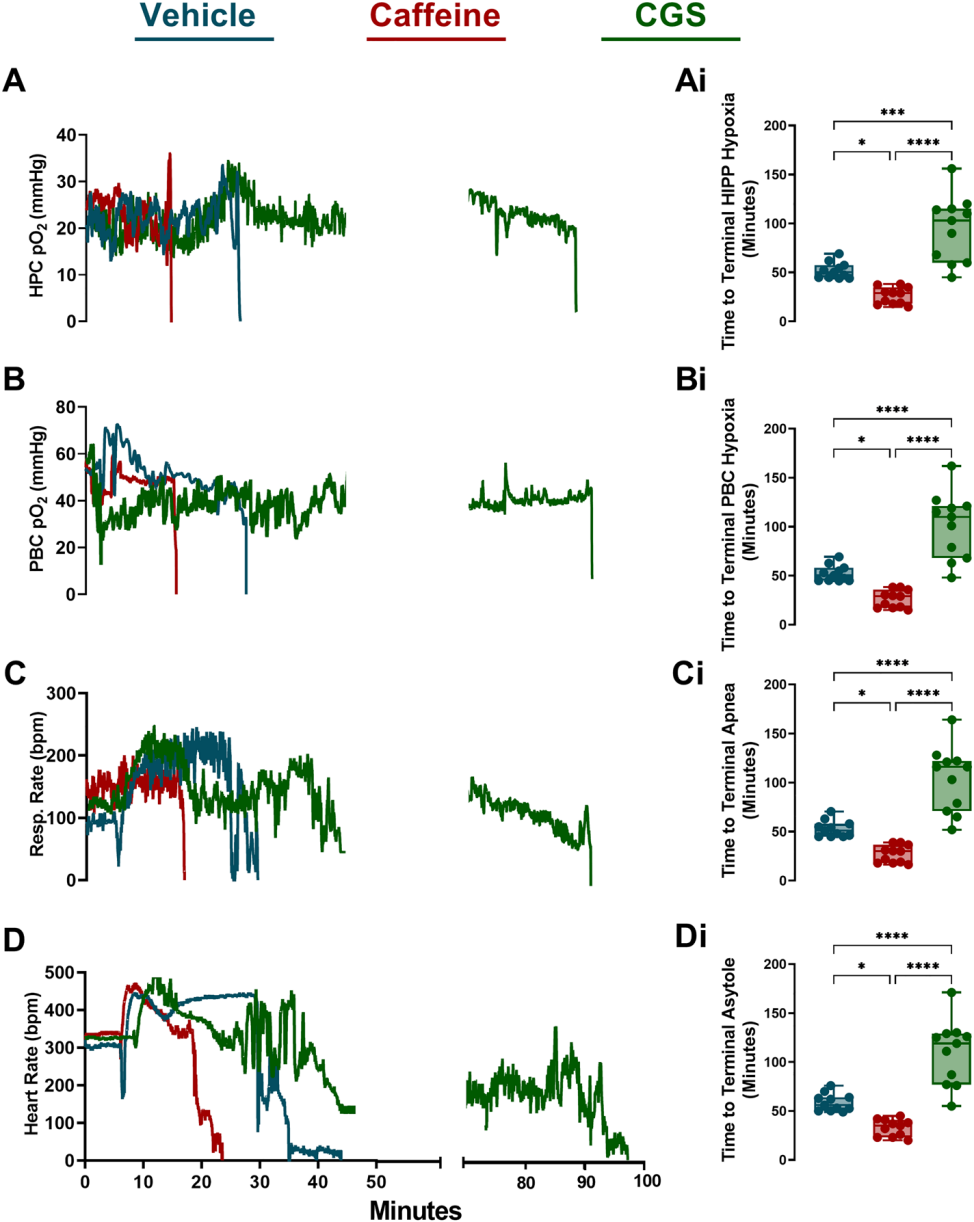
Acute caffeine administration decreases time to terminal apnea hypoxia and death. Representative profile from three C57BL/6J + kainic acid mice. (**A**,**Ai**) The mean time to terminal hypoxia of the hippocampus (HPC) was 51.93 ± 2.47 in vehicle (n = 11), 26.22 ± 2.58 in caffeine (n = 11) and 94.45 ± 10.11 in CGS-21680 (n = 11) mice ANOVA F (2,30) = 30.98, p =  < 0001***. (**B**,**Bi**) Mean time to terminal hypoxia of the pre-Bötzinger complex (PBC) was 52.32 ± 2.49 in vehicle, 26.37 ± 2.70 in caffeine and 101.2 ± 10.10 in CGS-21680 mice ANOVA F (2,30) = 37.46, p =  < 0.001****. (**C**,**Ci**) Mean time to terminal apnea in was 52.80 ± 2.52 in vehicle, 27.21 ± 2.65 in caffeine and 103.4 ± 10.01 in CGS-21680 mice ANOVA F (2,30) = 39.67, p =  < 0.0001****. (**D**,**Di**) Mean time to terminal asystole was 58.70 ± 2.69 in vehicle, 32.91 ± 2.61 in caffeine and 109.6 ± 9.92 in CGS-21680 mice ANOVA F (2,30) = 40.49, p =  < 0.0001****. Box plots represent mean ± SEM.

We have demonstrated that caffeine significantly reduces baseline hippocampal and brainstem oxygen while CGS-21680 significantly increases its levels. Our next step was to establish the sequence of events that occur with cardiorespiratory function and oxygen following the administration of either caffeine or CGS-21680. Following the intrahippocampal administration of kainic acid in C57BL/6J mice, the partial pressure of oxygen (pO_2_) remained above 10 mmHg. Oxygen levels at or below this threshold are considered severely hypoxic and are associated poor clinical outcomes leading to increased morbidity and mortality^25^. Vehicle treated mice displayed the following sequence of events leading to death: terminal electrographic seizure in the PBC, with behavioural bilateral tonic–clonic seizure, terminal hypoxia in the HPC, followed terminal hypoxia in the PBC with concomitant terminal apnea occurring once pO_2_ dropped below 4.8 ± 1.84 mmHg and finally followed by asystole several minutes later. Mice that were treated with caffeine displayed the same sequence of events, however, it was temporally accelerated with apnea occurring once pO_2_ dropped bellow 5.1 ± 2.18 mmHg. Mice that were pretreated with CGS-21680, displayed significantly longer latency to time of death compared to vehicle and caffeine mice (Fig. 4).

### Chronic adenosine receptor modulation influences time to death in Kcna1^−/−^ mice

**Figure 5:**
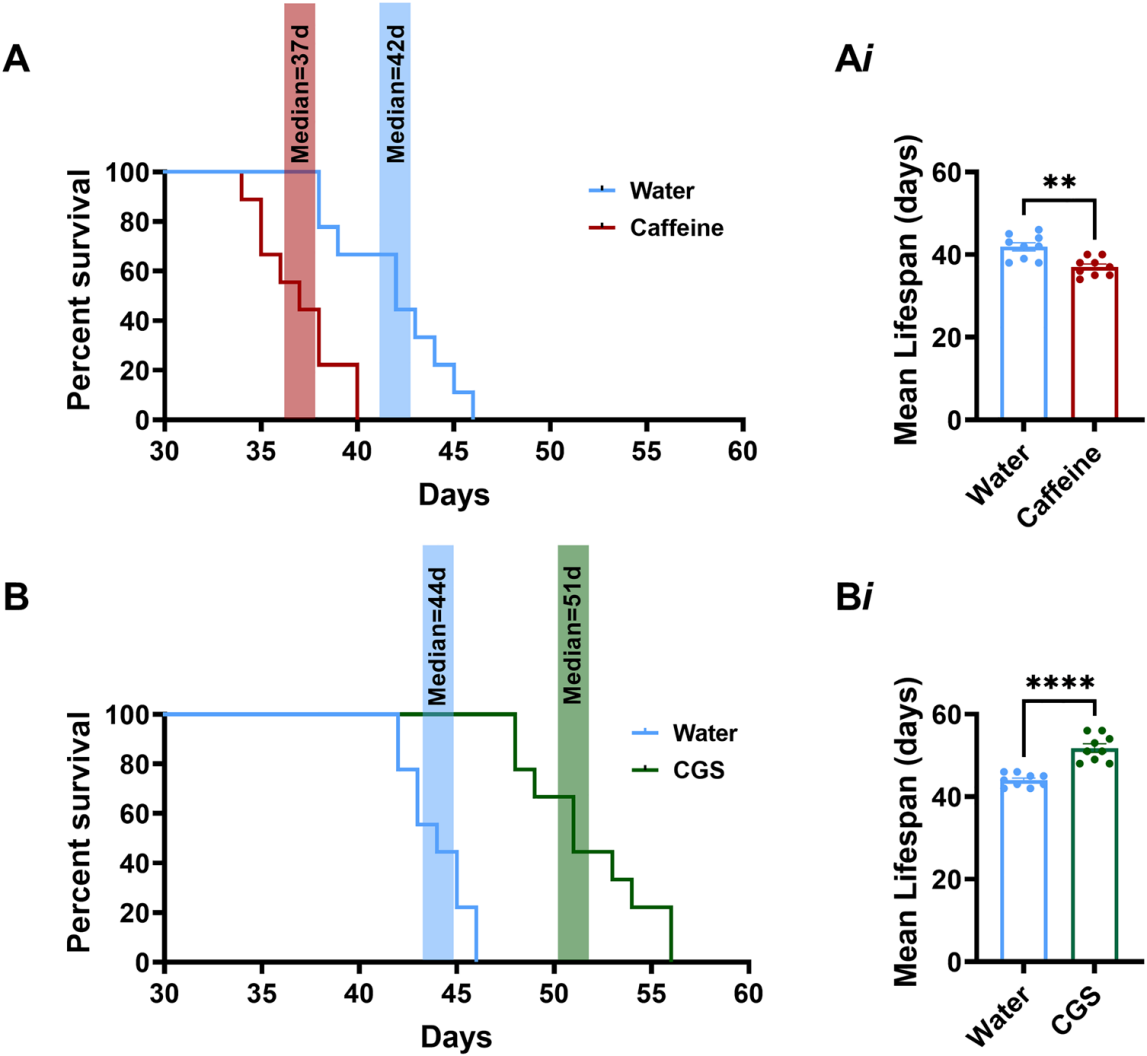
Chronic caffeine accelerates time to death and chronic CGS-21680 delays death. (**A**,**B**) (**A**) *Kcna*1^−*/*−^ mice treated with caffeine (n = 9) lived significantly shorter compared to vehicle (n = 9) treated mice. Median survival day for vehicle mice was 42d compared to median survival of caffeine treated of 37d p = 0.0015** (Log-rank test) (**Ai**). Mean survival day for vehicle treated mice was 41.89 ± 0.99 compared to mean survival of caffeine treated mice 37 ± 0.72, t_16_ = 3.97, p = 0.0011**. Chronic CGS-21680 reduces premature mortality and extends life, while chronic caffeine increases premature mortality. (**B**) *Kcna*1^−*/*−^ mice treated with CGS-21680 (n = 9) lived significantly longer compared to vehicle (n = 9) treated mice. Median survival day for vehicle mice was 44d compared to median survival of CGS-21680 of 51d ****p =  < 0.0001 (Log-rank test), (**Bi**) Mean survival day for vehicle treated mice was 44 ± 0.52 compared to mean survival of CGS-21680 treated mice 51.78 ± 1.05, t_16_ = 6.61, p =  < 0.0001****. Histobars represent mean ± SEM.

Our acute intrahippocampal kainic acid experiments indicated that a single dose of caffeine or its metabolite significantly accelerated time to death and CGS-21680 reduced time to death. Here we determined if the observations made in the acute experiments could be replicated chronically using *Kcna1*^−*/*−^ mice which displays frequent self-generated seizures beginning in early life through to adulthood with high incidence of premature seizure-induced death^26^. On postnatal day (P) 30 mice were randomly assigned to either caffeine-in-water or water-only groups. The median survival time was significantly shorter in mice treated with caffeine (n = 9) compared to the water-only (n = 9) group (P37 vs. P42, respectively). Our acute experiments also indicated that a single dose of the A_2A_ agonist, CGS-21680, significantly extended life. To test whether chronic CGS-21680 could also produce significant life extension, we administered this drug chronically. Due the solubility limitations, we administered CGS-21680 intracerebroventricularly via an osmotic minipump. The median survival time was significantly longer in mice treated with CGS-21680 (n = 9) compared to the water only (n = 9) group (P51 vs. P40 respectively) (Fig. 5). These data clearly demonstrate that chronic ingestion of caffeine may be detrimental in those with epilepsy, increasing the likelihood of premature mortality. Conversely, the A_2A_ agonist CGS-21680 was shown to be neuroprotective.

## Discussion

Self-limited seizures in both rodents and people with epilepsy result in vasoconstriction, hypoperfusion and hypoxia^12–14^. Postictal hypoxia was hypothesised to lead to SUDEP^16^. George and colleagues (2023) recently tested this neurovascular based hypothesis of SUDEP and found that seizure activity in brainstem breathing centres led to severe hypoxia and subsequent breathing failure^17^. They also identified two drug targets, cyclooxygenase-2 and L-type calcium channels, that when blocked prevented postictal hypoxia for a period of time and extended life in their models of seizure-induced death. Caffeine and other drugs that modulate adenosine receptors were previously found to increase the length of postictal hypoxia^18^. In the present study we observed that caffeine significantly lowers baseline oxygen in the hippocampus and brainstem and accelerates time to death. We provide evidence against caffeine’s involvement of the A_1_ receptor by using the selective A_2A_ agonist CGS-21680 which was able to increase baseline oxygen and decrease time to death. Our study is the first to show an association between caffeine and postictal hypoxia in SUDEP pathophysiology.

We demonstrated that caffeine accelerated time to death in both our acute and chronic models of seizure-induced death. This study made the critical observation that acute dosing of caffeine significantly reduced baseline physiological oxygen in both the hippocampus and pre-Bötzinger complex. The neurovascular theory of SUDEP posits that seizures that spread to the brainstem lead to hypoxia of breathing centres and subsequent breathing failure. The pre-seizure reduction in oxygen seen in response to caffeine can serve to increase the likelihood of fatal outcomes as it sets the stage for death. The cerebral vasoconstrictive effects of caffeine are well established^27–29^. Caffeine exerts its effect as a vasoactive substance on a wide range of molecular targets^30^. Primarily, it acts as a non-selective antagonist of the A_1_ and A_2A_ adenosine receptors. We first isolated the receptor subtype responsible for promoting caffeine’s effect on postictal hypoxia and early death in our models. We first examined A_1_ activation using the selective A_1_ agonist N6-Cyclopentyladenosine (N6). In our hands, N6 showed a similar effect to caffeine by accelerating time to death. We used the A_2A_ selective agonist CGS-21680 which reduced time to death in both our acute and chronic models. Based on these receptor pharmacology results, we have evidence that caffeine is likely interacting with A_2A_ receptors to produce its vascular effects.

Contrary to our findings, a previous study in mice showed that acute caffeine administration increased survival in a model of seizure-induced death^31^. In their study, to identify if increased adenosine was responsible for SUDEP, caffeine was administered after seizure onset following systemic kainic acid administration. Their study however did not quantify physiological parameters such as respiratory and cardiac physiology which limits the ability to draw conclusions regarding the cause of death. Further, the caffeine dose of 40 mg/kg exceeds the equivalent dose consumed by a typical human, reducing clinical translatability^32^.

The present study utilized two previously published models of seizure-induced death: acute intrahippocampal kainic acid which causes several bouts of bilateral tonic–clonic seizures with a resultant terminal seizure, and chronic *Kcna1*^−*/*−^ mice that display self-generated bilateral tonic–clonic seizures early in life with a high incidence of seizure-induced death^17^. These models were chosen as they recapitulate core features of SUDEP in humans, including young age, high seizure burden, and bilateral tonic–clonic seizures. As a function of these all-or-none seizure-induced death models, all mice died regardless of treatment as these drugs were administered to assess how they modulate time to death. We recorded several physiological parameters to assess the sequence of events leading to death. Breathing in this study was measured using a diaphragmatic EMG which records phrenic nerve activity. The EMG method determines cessation of diaphragmatic contraction and does not measure ventilation. Therefore, we cannot rule out a potential peripheral obstruction such as a laryngospasm. This model also produces several bouts of recurrent seizures which can lead to metabolic derangements and contribute to premature death. In our chronic study, *Kcna1*^−*/*−^ mice that received caffeine or CGS-21680 did not have their seizures recorded which limits our ability to assess whether latency to death was mediated by anti-seizure effects.

The intrahippocampal kainic acid model provides a unique tool to examine the physiological alterations that occur during and after a seizure, leading to mortality. High doses of kainic acid can evoke status epilepticus (SE)^33^ which precludes a diagnosis of SUDEP. However, lowering the dose can produce non-status models^34,35^. Previous reports have established cerebral oxygen markers of SE in rodent models. Using oxygen sensitive probes, it has been demonstrated that SE produces robust and long-lasting hyperoxia which correlates with increased cerebral blood flow^36,37^. This is also consistent with other reports that show sustained cerebral hyperperfusion following kainic acid-induced SE^38^ and pilocarpine- induced SE^39,40^. During recordings in this study, all mice had at least two behavioral seizures followed by postictal hypoxia and severe hypoxia prior to death, which is not associated with SE.

Brain function depends critically on moment-to-moment regulation of oxygen supply by the bloodstream to meet changing metabolic needs as seen during seizures. In fact, seizures significantly increase metabolic rate and oxygen consumption by mitochondria, severely limiting the availability of oxygen within the system^41^. This metabolic reduction in oxygen could contribute to seizure-induced mortality if it involved the brainstem and was temporally correlated to postictal vasoconstriction. Recently, Villa and colleagues (2023) demonstrated that postictal mitochondrial dysfunction and the unchecked conversion of oxygen to reactive oxygen species also contributes to postictal severe hypoxia^42^. Further they found that using a mild mitochondrial uncoupler which prevents the conversion of oxygen to reactive oxygen species ameliorated postictal hypoxia and provide a potential therapeutic treatment to reduce the risk of SUDEP^42^.

The endogenous nucleoside adenosine plays an important role in several physiological processes^43^. In the central nervous system, adenosine imparts an inhibitory tone on synaptic activity and is a potent modulator cerebral blood flow and tissue oxygenation^43–45^. Adenosine levels in the brain increase during pathological states such as ischemia, hypoxia, and seizures^46,47^ and may play a role in seizure termination. Despite its antiseizure properties, excessive adenosine is postulated to exert detrimental effects in the brainstem^48,49^. The adenosine hypothesis of SUDEP states that seizure-induced increase of adenosine in the brainstem leads to inhibition of breathing circuits via the activation of adenosine receptors^31,48,49^. However, contrary to this hypothesis, we show that activation of the A_2A_ receptor is beneficial by extending life. The endogenous ligand for the A_2A_ receptor is adenosine. Among the adenosine receptors, A_2A_ is the primary receptor responsible for the vasodilatory effects of adenosine.

We also demonstrate that the A_2A_ agonist CGS-21680 both increased baseline hippocampal and brainstem oxygen and prolonged time to death. Activation of the A_2A_ receptor mediates vasodilation in a variety of vascular beds including cerebral vasculature^50–54^. This work aligns with previous studies showing a robust dilatory effect of CGS-21680^50^. Ngai and colleagues (2001) found that CGS-21680 induced significant dose-dependent vasodilation of cerebral arterioles^55^. Further, using the selective A_2A_ antagonist ZM-241385, they were able to inhibit the dilatory response to both CGS-21680 and adenosine. They concluded that the A_2A_ receptor was responsible for the adenosine-induced dilatory effect they saw in rat cerebral arterioles.

Caffeine has been shown to display vasoactive properties through a number of molecular systems, including adenosine^30^. Adenosine’s actions are mediated by specific cell surface receptors coupled to G proteins classified as A_1_, A_2A_, A_2B_, and A_3_^56^. Caffeine acts as a competitive antagonist of the A_1_ and A_2A_ receptors. However, caffeine also influences cerebrovascular dynamics through mechanisms independent of adenosine receptors. Caffeine can liberate calcium from intracellular stores and modulate calcium channels and increase prostaglandin E2 (PGE2) synthesis^57–59^. Previous studies have found that seizure-induced vasoconstriction is mediated by vasoactive prostanoids, such as PGE2^12,16^. They found that ibuprofen, a cyclooxygenase-2 blocker prevented postictal vasoconstriction, hypoperfusion and brain tissue hypoxia. They also showed that ibuprofen did not influence seizure duration, indicating that its effects were attributable to the neurovascular phenomenon and not a seizure-altering mechanism^17^. Caffeine’s ability to increase the production of PGE2 coupled with seizure induced PGE2 activation could potentially culminate to produce fatal outcomes when it occurs in the brainstem and may have occurred in our models.

Sudden unexpected death in epilepsy continues to be a major cause of death in people with drug resistant epilepsy^60,61^. Our data support a detrimental effect of caffeine on SUDEP pathophysiology in two models of seizure-induced death. In our hands acute administration of caffeine significantly decreased oxygen in both the hippocampus and brainstem and accelerated time to death. The ubiquitous nature of caffeine consumption makes this finding of considerable clinical significance. Caffeine consumption is an easily modifiable risk factor for SUDEP and reveals a promising strategy to reduce death. Additionally, the present study points to the utility of A_2A_ agonists as a promising tool to reduce SUDEP risk.

## Methods

### Animals

Animal care and use procedures were carried out in accordance with standards set by the Canadian Council for Animal Care and ARRIVE guidelines. Experimental protocols were approved by the Health Sciences Animal Care Committee at the University of Calgary (AC20-0170). Male C57BL/6J mice aged 7 weeks (P50) and weighing 25–32 g were obtained from Charles River Laboratories (Wilmington, MA, USA). Male *Kcna1*^−*/*−^ mice on a C3HeB/FeJ background were obtained from heterozygous breeding colonies established at the University of Calgary. Animals were housed in a temperature-controlled environment with ad libitum access to food and water. All procedures complied with ARRIVE guidelines.

### Acute experiment surgeries

#### Brain implant surgery

Male C57BL/6J mice on postnatal day 50 were anesthetized with 5% isoflurane and maintained on 2% isoflurane mixed with 100% oxygen. The skull was secured in a stereotaxic apparatus (David Kopf Instruments) using ear and incisor bars. Diluted lidocaine (0.5% 1:4 lidocaine, saline) was injected subcutaneously over the incision site followed by a sagittal incision along the midline of the head. Burr holes were drilled in the skull at stereotaxic coordinates. Bipolar depth electrodes were implanted unilaterally into the dorsal hippocampus at these coordinates relative to bregma: (AP) − 2.2, (ML) − 2.5, (DV) − 2.0 and the pre-Bötzinger complex at: (AP) − 7.0, (ML) − 1.4, (DV) − 4.1. Oxygen sensing optodes (Oyxylite, Oxford Optronix) were implanted in the ipsilateral ventral hippocampus at: (AP) − 1.5, (ML) − 0.8, (DV) − 1.5 and in the contralateral pre-Bötzinger complex at: (AP) − 7.0, (ML) + 1.4, (DV) 4.1. A 22-gauge infusion guide cannula (Plastics One, Roanoke, VA) was implanted in the contralateral dorsal hippocampus at: (AP) − 2.7, (ML) + 3.0, (DV) − 3.0. Electrodes and probes were secured to the skull using Metabond Quick Adhesive (C&B) and dental acrylic. Mice were administered injectable buprenorphine (0.05 mg/kg) every 12 h for 3 days post-operatively and mice recovered for 3 days before diaphragmatic surgery.

#### Diaphragmatic surgery

On the same day after brain implantation surgery, mice were maintained on 2% isoflurane mixed with 100% oxygen. Diluted lidocaine (0.5% 1:4 lidocaine, saline) was injected subcutaneously at the incision site on the dorsal segment of the ribs just below the ribcage. Electrode wires were implanted as described by Pagliardini and colleagues into the diaphragm to record phrenic nerve activity^62^. In brief, multi-stranded Teflon-coated stainless-steel electrode wire (#AS633, Cooner Wire) were passed through the diaphragm using a suture needle and secured in place by a knot. A second electrode wire was placed 1 mm away from the first to provide a bipolar recording. The electrode ends were then tunneled under the skin to an incision on the animal’s dorsum, between the scapulae. The uninsulated ends of the electrode wires were then attached to male amphenol pins and secured in a 9-pin ABS socket (Ginder Scientific) that protruded from the back. The incision sites were sutured, and the mice recovered. Mice were administered injectable buprenorphine (0.05 mg/kg) every 12 h for 3 days post-operatively and were given 7 days to recover before beginning acute experiments.

### Chronic experiment surgery

#### Intracerebroventricular surgery

Male *Kcna1*^−*/*−^ mice on postnatal day 26, mice were anesthetized with 5% isoflurane and maintained on 2% isoflurane mixed with 100% oxygen. The skull was secured in a stereotaxic apparatus using ear and incisor bars. Diluted lidocaine (0.5% 1:4 lidocaine, saline) was injected subcutaneously over the incision site followed by a sagittal incision along the midline of the head. The incision was then extended over the dorsum of the mouse posterior to the scapulae. Haemostats were introduced subcutaneously to widen incision and produce dead space for pump insertion. A burr hole was made over the right lateral ventricle at these coordinates relative to bregma: (AP) + 0.3, (ML) − 1.0, (DV) − 3.0^30^. The infusion cannula was secured to the skull using Metabond Quick Adhesive (C&B) and dental acrylic. Mice were administered injectable buprenorphine (0.05 mg/kg) every 12 h for 3 days post-operatively and mice were allowed to recover for 3 days before starting the experiment on P30. The patency and placement of the cannula was verified at the end of the experiments using methylene blue dye (Sigma-Aldrich) administration.

### Drug administration

#### Acute drug administration

After a 7-day recovery period from surgery, male C57BL/6J mice were administered either caffeine (10 mg/kg), theophylline (10 mg/kg), N6-Cyclopentyladenosine (N6) (1 mg/kg) or CGS-21680 (1 mg/kg) or vehicle DMSO for lipophilic drugs or saline for hydrophilic drugs) intraperitoneal (i.p) 30 min prior to infusion of kainic acid. Kainic acid (1.4 μg in 0.4 μL) was infused unilaterally through an intrahippocampal cannula into the right dorsal hippocampus at 0.1 μL/min through a 1 μL Hamilton syringe (Hamilton Robotics, Reno NV) and micro-syringe pump (Harvard Apparatus, model 55-2222, Canada). All drugs were purchased from Cayman Chemicals. In acute experiments, local field potential, EMG and brain pO_2_ were recorded continuously and time to death following infusion was recorded.

### Chronic caffeine in drinking water

Male *Kcna1*^−*/*−^ mice on a C3HeB/FeJ congenic background were used. These mice carry a deletion of the *Kcna1* gene on chromosome 6 and are lacking functional voltage-gated Kv1.1 channels. Starting the experiment on postnatal day 30, male mice received either water (vehicle) or water with 0.5 mg/mL caffeine (Sigma-Aldrich). Mice were monitored every 12 h and date of death recorded. Caffeine was protected from light and replaced every 12 h to ensure photostability and solubility.

### Chronic intracerebroventricular CGS-21680 administration

Male *Kcna1*^−*/*−^ mice were implanted with Alzet 42-day mini-pumps (model 2006; Durect, Cupertino) which continuously delivered CGS-21680 (or vehicle) into the left lateral ventricle (0.2 mg/mL, pumping rate 0.15 μL/h) this dose was selected based on pilot data suggesting toxicity and lethality at higher concentrations. CGS-21680 was dissolved in DMSO, PEG and 100% ETOH (50:40:10) (Cayman Chemicals). Mice were monitored every 12 h and date of death recorded.

### Physiological recordings

#### Absolute oxygen recordings

Absolute oxygen recordings were measured using an implantable fibre-optic oxygen-sensing probe (Oxylite, Oxford Optronics). These probes emit a short burst of LED light to a platinum fluorophore at the tip of the probe. When oxygen molecules collide with the fluorophore the light emitted by the probe is quenched and is registered as a decay in luminescence which is relayed to the terminal for display. The probe allows for accurate and continuous readings of oxygen in brain parenchyma in the awake and freely moving animal. The optode can measure a volume of 500 µm^3^ between 0 and 90 mmHg. Unless otherwise stated, sample rate of 60 light pulses/min were used at 1 Hz. Severe hypoxia is defined as < 10 mmHg because oxygen levels at or below this threshold are associated with brain injury resulting from cellular damage and death leading to worse clinical outcomes.

### Electromyography recordings and analysis

The EMG was analyzed using previously published methods^14^. Heart rate and respiratory rate was obtained from raw EMG signal as a post-processing procedure. To obtain respiratory rate, the signal was low pass filtered below 0.01 Hz using a fifth-order Butterworth filter to remove signal drift and to determine during which part of the recordings the mouse was alive. Next, the raw EMG data was again low pass filtered below 4 Hz to extract respiratory information. The duration of the recording while the mouse was alive was the analyzed for individual breaths, done by applying a 1D data peak detection algorithm (peakutils.peak). Local maxima larger than a normalized threshold height were identified as breaths (the exact amplitude being dependent on the signal strength in the individual dataset). Heart rate data was processed by using custom algorithm (Python 2.7.12, scipy 0.18.1, peakutils 1.1.0). The signal was low pass filtered at 5 Hz using a fifth-order Butterworth filter to extract respiration and movement artifact. This low frequency signal was subtracted from the raw EMG to remove low frequency components, leaving only high frequency components such as heartbeats and signal noise. Individual heartbeats were determined by identifying local maxima using a 1D data peak detection algorithm (peakutils.peak). Only peaks over a certain threshold amplitude defined relative to the maximum amplitude in the signal (the exact threshold being dependent on the overall signal strength in the individual dataset) were accepted as heartbeats. A minimum distance between peaks was defined as 12 ms for heartbeats, as heartbeats at a higher frequency would not be physiologically possible. Breathing and heart rate were obtained by calculating the time difference between peaks and creating a moving average across the entire data set.

### Statistics

All statistics were calculated using GraphPad Prism version 9 (GraphPad Software, Inc., San Diego, CA, USA, 2003) Unpaired (comparisons between animals) t-tests were used for experiments with only two groups. When more than two groups were compared either a one-way ANOVA, or two-way ANOVA were applied. Survival curves were analyzed by Kaplan–Meier survival estimates using the log-rank test. A priori normality testing (D’ Agostino and Pearson) was followed by a student’s t-test. Significance thresholds were set to: *p < 0.05. Unless otherwise stated, values were expressed as means ± standard error of the mean (SEM).

## Data Availability

The data underlying this article will be shared on reasonable request to the corresponding author.
